# Flow-cytometry phenotypic assessment of immune cell subsets reflecting function for the identification of breast cancer patients receiving vaccine plus docetaxel with longer progression-free survival

**DOI:** 10.1186/2051-1426-1-S1-P51

**Published:** 2013-11-07

**Authors:** Italia Grenga, Renee N Donahue, James L Gulley, Christopher Heery, Ravi A Madan, Jeffrey Schlom, Benedetto Farsaci

**Affiliations:** 1 Laboratory of Tumor Immunology and Biology, CCR, NCI, NIH, Bethesda, MD, USA

## Purpose

Aim of this study was to assess whether specified immune cells subsets at baseline could help identifying patients with longer progression-free survival (PFS) in a clinical trial of metastatic breast cancer patients receiving docetaxel±vaccine.

## Methods

We applied flow-cytometer analysis of PBMCs harvested before treatment from patients (n=43) enrolled in a small randomized phase II study of docetaxel alone (n=20) or in combination with PANVAC^TM^-V (Vaccinia) and PANVAC^TM^-F (Fowlpox) encoding for the tumor-associated antigens CEA and MUC-1, along with a TRIad of COstimulatory Molecules (B7-1, ICAM-1, and LFA-3; called TRICOM) (n=23). As criterion 1, we analyzed the frequency standard immune subsets, i.e. CD4, CD8, NK, Treg, MDSC, and their ratios. As criterion 2, we measured phenotypes indicating immune function, i.e. central memory T lymphocytes, T cells expressing at ≥2 suppressive markers among CTLA-4, PD1, TIM3, and 2B4, CD49d^-^ Tregs, lin^-^ MDSCs, CD56^br^CD16^-^ NK cells, and their ratios. An immunoscore was generated based on the analysis of tertiles. Log-Rank Kaplan Meier analysis was applied to evaluate differences of PFS between patients with low- and- high immunoscore.

## Results

The predetermined immunoscore based on phenotypes indicating immune function allowed discrimination between patients with longer PFS vs. shorter PFS in vaccine plus docetaxel arm (p<0.001, HR=0.049) but not in docetaxel alone arm (p=0.875; HR=0.926).

## Conclusions

The calculation of an immunoscore based on a flow-cytometer screening of predetermined immune subsets indicating immune function from PBMCs before treatment could be a potential useful tool for the identification of patients that can benefit from combination immunotherapy.

